# Validation of phylogenetic signals in amplified fragment length data: testing the utility and reliability in closely related taxa

**DOI:** 10.1186/1756-0500-2-26

**Published:** 2009-02-25

**Authors:** Jessica A Wooten, Lori R Tolley-Jordan

**Affiliations:** 1 The University of Alabama Department of Biological Sciences, Box 870344, Tuscaloosa, AL 35487, USA

## Abstract

**Background:**

Discriminating taxa with the nuclear marker, amplified fragment length polymorphism (AFLP) has been accomplished for various organisms in economic, ecological, and evolutionary studies. The protocol available for AFLP generation does not require prior knowledge of the genome; however, it is often extensively modified to fit the needs of the researcher. Modification of this protocol for new labs is intimidating and time-consuming, particularly for taxa in which AFLP have not been previously developed. Furthermore, determining what constitutes quality output during different stages of fragment generation is not well defined and this may further hinder the use AFLP by new researchers.

**Findings:**

We present a step-by-step AFLP protocol, using flourophore-labeled primers for use with automated sequencers, including examples of both successful and unsuccessful results. We sufficiently normalized peak intensity and standardized allele calling across all samples for each primer combination. Repeatability was assessed with a phylogenetic tree in which replicate samples clustered together using the minimum evolution procedure. We found differences greater than 10% in allele position among replicated samples would cause replicates to no longer cluster. To minimize offset allele positions, we suggest that researchers analyze different primer combinations at the same time using multiple dyes with the automated sequencer to minimize mismatched alleles across replicates.

**Conclusion:**

For researchers wanting to use AFLP, this molecular technique is difficult and time-consuming to develop. Clarifying what constitutes quality output for each step in AFLP generation will help to reduce redundant trials in protocol development and, in turn, advance the discipline of population genetics.

## Background

Amplified fragment length polymorphism (AFLP) has been extensively used to investigate population genetics [1,2], genome mapping [3,4], and genetic structure of intra- and interspecific taxa [5-7], especially in plants, microbes, and fungi, but less often for animal taxa [8]. This method has many benefits over other genetic techniques for addressing questions in population genetics including: low start-up cost, high repeatability, the ability to assay a large number of polymorphic loci in many individuals in a relatively short period of time, and no prior knowledge of the genome or sequence data is necessary [8-14]. Using the original AFLP protocol [9,15], an investigator generates between 50 – 100 restriction fragments, which are generally less than 600 base pairs (bp), per primer combination, when fragments are amplified and detected on denaturing polyacrylamide gels. Technological advancements (e.g., automated capillary sequencers and flourophore-labeled primers) have lead to reduced scorer bias, increased the overall number of fragments that may be scored with confidence, and promoted analyses of larger sample sizes [16].

There is a standard protocol available for AFLP generation [9] that has been modified by researchers for their specific study taxa [8]. However, modification of this protocol [9] for new labs is intimidating and can be time-consuming to develop for taxa in which AFLP have not been previously established. Even after successful AFLP fragment generation, determining what constitutes quality AFLP output (i.e., electropherograms) is unclear. Although software is available for analyzing electropherograms generated from automated capillary sequencers (e.g., GeneMapper or GeneMarker), there is a lack of clearly refined protocols to assess the quality of generated AFLP fragments. Thus, understanding and interpretation of successful results can be challenging to researchers first using AFLP and redundant trials in protocol development can hinder advancement of research. Because of these challenges, the objectives of this study were to: (1) provide guidelines for AFLP generation with automated analyses using a protocol amenable for disparate animal groups; (2) construct procedures to normalize and standardize AFLP electropherograms; and (3) test the repeatability of AFLP samples processed with an automated capillary sequencer and analyzed with applicable software.

### Study Animals

**Figure 1:**
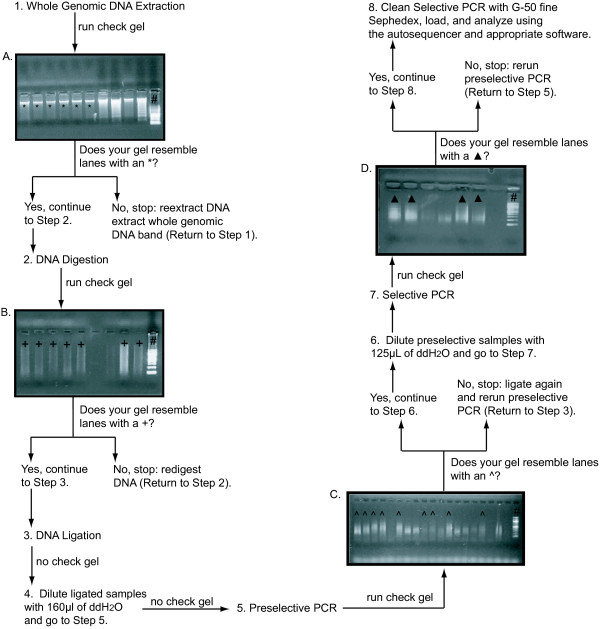
**Step-by-step protocol for AFLP generation**. Schematic representation of each step in the AFLP protocol represented on 1% sodium borate agarose gels. All gel images were generated from undiluted DNA solutions. Each gel image contains lanes that represent acceptable products as indicated by a unique symbol. The 100 bp ladder is denoted by # in all gel images.

Snails (*Elimia*) and salamanders (*Desmognathus*) were used to evaluate the standardized AFLP protocol. Two different DNA-extraction techniques were employed to achieve high-quality, whole genomic DNA. The DNA was considered high quality if there was an optical density (OD) of 260/230 between 1.8 – 2.1 and 260/280 between 1.8 – 1.9 using a spectrophotometer (NanoDrop ND – 1000). Each sample was visually inspected on 1% sodium borate agarose gels (Figure 1a).

For snails, whole genomic DNA was extracted from head tissue using a modified protocol [17]. Each snail head was placed in a 600 μl CTAB solution (2% CTAB, 1.4 M NaCl, 20 mM EDTA, 100 mM Tris – HCl pH 8, 0.2% β-mercaptoethanol) to which 15 μl of 10 mg/ml Proteinase K was added followed by incubation at 55°C for 3 h. Each sample was washed twice with 600 μl of chloroform:isoamyl alcohol (24:1) and allowed to precipitate overnight in cold isopropyl alcohol. Precipitated DNA was purified with 95% ethanol and a final 70% ethanol wash. Samples contained a strong band of whole genomic DNA, but also contained degraded DNA and RNA that negatively affect the quality of downstream reactions [15]. Thus, all samples were further purified using a QIAEX II Gel Extraction Kit (Qiagen, Calencia, CA). This procedure yielded between 5 – 75 ng of high-quality DNA.

For salamanders, whole genomic DNA was extracted from approximately 5 mm of tail tissue from each individual using a DNeasy Kit protocol for animal tissues (Qiagen, Valencia, CA). This protocol yielded between 5 – 100 ng of high-quality, whole genomic DNA.

### Amplified Fragment Length Polymorphism and Primer Screening Protocol

All enzymes and restriction buffers were obtained from New England Biolabs, unless otherwise noted. Digestion reactions in 20 μl volumes were performed on whole genomic DNA with a concentration ranging from 10 – 70 ng/μl following cocktail per reaction: 2 μl *Eco*RI 10× restriction buffer, 1.0 μl *Eco*RI (20,000 U/ml), 0.2 μl *Mse*I (10,000 U/ml; in snails, 0.8 μl *Mse*I was used), and 12 μl H_2_O. Following a 5 h incubation period at 37°C (in snails, 3 h); we ensured adequate digestion using a 1% sodium borate agarose gel (Figure 1b). Before ligation, double-stranded adaptor pairs (10 mM) were constructed from the complementary single-stranded oligonucleotides (Table 1). These adaptor pairs were joined by combining 250 μl of each adapter and heating the solution at 95°C for 5 min and then cooled to 25°C (Table 2). Ligation proceeded using a 20 μl mixture consisting of 12 μl H_2_O, 4 μl 10× T4 DNA ligase buffer, 1.5 μl of the *Eco*RI and *Mse*I adapter pairs (75 pmols), and 1.0 μl of T4 DNA ligase to each digestion solution. These samples were incubated for 10 h (in snails, for 12 h) at 16°C. Following ligation, each sample was diluted with 160 μl H_2_O.

**Table 1:** Adapter and primer sequences used in AFLP.

**Adapter Sequence**		
*Eco*RI-Adapter	5'-CTCGTAGACTGCGTACC**AATTC**-3' 3'CATCTGACGCATGG**TTAAG**-5'	

*Mse*I-Adapter	5'-GACGATGAGTCCTGAG**TAA**-3' 3'-TACTCAGGACTC**ATT**-5'	

		

**Snails**	***Eco*RI Sequence (5'-3')**	***Mse*l Sequence (5'-3')**

Preselective Primer 1	CTCGTAGACTGCGTACCAATTCGAC***CA***	GACGATGAGTCCTGAGTAAGAC***CG***

Preselective Primer 2	CTCGTAGACTGCGTACCAATTCGAC**CA**	GACGATGAGTCCTGAGTAAGAC***GC***

Preselective Primer 3	CTCGTAGACTGCGTACCAATTCGAC***CT***	GACGATGAGTCCTGAGTAAGAC***CG***

Preselective Primer 4	CTCGTAGACTGCGTACCAATTCGAC***CT***	GACGATGAGTCCTGAGTAAGAC***GC***

		

Selective Primer 1	CTCGTAGACTGCGTACCAATTCGAC***CAA***	GACGATGAGTCCTGAGTAAGAC***CG***

Selective Primer 2	CTCGTAGACTGCGTACCAATTCGAC***CAA***	GACGATGAGTCCTGAGTAAGAC***GC***

Selective Primer 3	CTCGTAGACTGCGTACCAATTCGAC***CTG***	GACGATGAGTCCTGAGTAAGAC***CG***

Selective Primer 4	CTCGTAGACTGCGTACCAATTCGAC***CTG***	GACGATGAGTCCTGAGTAAGAC***GC***

		

**Salamanders**		

Preselective Primer 1	CTCGTAGACTGCGTACCAATTCGAC***CA***	GACGATGAGTCCTGAGTAAGAC***GA***

Preselective Primer 2	CTCGTAGACTGCGTACCAATTCGAC***CA***	GACGATGAGTCCTGAGTAAGAC***GC***

Preselective Primer 3	CTCGTAGACTGCGTACCAATTCGAC***CA***	GACGATGAGTCCTGAGTAAGAC***CG***

Preselective Primer 4	CTCGTAGACTGCGTACCAATTCGACAC*	GACGATGAGTCCTGAGTAAGACCA*

		

Selective Primer 1	CTCGTAGACTGCGTACCAATTCGAC***CAA***	GACGATGAGTCCTGAGTAAGAC***CA***

Selective Primer 2	CTCGTAGACTGCGTACCAATTCGAC***CAA***	GACGATGAGTCCTGAGTAAGAC***GC***

Selective Primer 3	CTCGTAGACTGCGTACCAATTCGAC***CAA***	GACGATGAGTCCTGAGTAAGAC***CG***

Selective Primer 4	CTCGTAGACTGCGTACCAATTCGACACC*	GACGATGAGTCCTGAGTAAGACCA*

Adapter and preselective and selective primer sequences for the rare cutter, *Eco*RI and the frequent cutter, *Mse*I. Black text indicates the core sequence of either of the adapter, *Eco*RI or *Mse*I sequences. **Black Bold **text indicates the restriction portion of the adapter sequence. ***Black Bold Italicized ***text indicates the preselective or selective primer sequence. The * indicates a primer combination used in the salamander portion of the study, but the results are not presented here.

**Table 2:** Cocktails used for preselective PCR in AFLP.

**Preselective Cocktail**	
**Reagent**	**Quantity (μl)**

15 pmol Preselective *Eco*RI Primer	1.2

15 pmol Preselective *Mse*l Primer	1.2

10 mM dNTP	4.0

Formamide	1.0

25 mM MgCl_2_	2.5

H_2_O	24.8

10× PCR Buffer	10.0

Taq DNA Polymerase	0.5

**Total**	45.2*
*Add 40 μl of the preselective cocktail to 10 μl diluted restriction-ligation product. The remaining 5.2 μl allows for pipetting error.	

Reaction cocktails for preselective PCR amplifications of the ligated DNA.	

We conducted a preselective primer screen to determine the efficacy of primer pairs. In the standardized protocol [9], only one base is added during preselective PCR. We tested *Eco*RI + NN, where N represents the number of additional base pairs attached to the core sequence (i.e., NN = two additional base pairs; Table 1), and *Mse*I + NN primer combinations (n = 256) by checking for high-quality bands on a 1% sodium borate agarose gel, since this was the most cost-effective means for determining primer efficacy. Thus, we used this two base extension for preselective PCR. Using the chosen primer combinations, we completed preselective PCR involving using the cocktail in Table 2 and the cycling conditions in Table 3. Preselective solutions were diluted with 125 μl and 160 μl of H_2_O in salamanders and snails, respectively. A 1% sodium borate agarose gel was used to visually check each sample (Figure 1c).

**Table 3:** Thermocycler conditions for preselective PCR.

**Preselective Cycles**			
**Step**	**Temperature**	**Duration**	**Number of Cycles**

Initial Denaturation	94°C	120 sec	1

			

Denaturation	94°C	50 sec	

Annealing	56°C	60 sec	2

Extension	72°C	60 sec	

			

Denaturation	94°C	50 sec	

Annealing	56°C	60 sec	25

Extension	72°C	120 sec	

			

Final Extension	72°C	120 sec	1

We ran a subsequent selective primer screen to determine which *Eco*RI + NNN and *Mse*I + NN would generate highest quality bands on a 1% sodium borate agarose gel. When labeling *Eco*RI with a flourophore (i.e., 6-FAM) for selective PCR, the label should always be attached to the 5' end and to any nucleotide except guanine because of the effects of guanine quenching [18]. Using the chosen primer combinations, we completed selective PCR using the cocktail in Table 4 and the cycling conditions in Table 5. A 1% sodium borate agarose gel was used to visually check each sample (Figure 1d). In general, if samples were visible on the 1% sodium borate agarose gel they are too strong for the autosequencer; thus, all samples were diluted between 25 – 50% with doubly-distilled water.

**Table 4:** Cocktails used for selective PCR in AFLP.

**Selective Cocktail**	
**Reagent**	**Quantity (μl)**

5 pmol Labeled Selective Eco RI Primer	1.5

15 pmol Unlabeled Selective Mse I Primer	1.5

10 mM dNTP	3.0

Formamide	0.5

25 mM MgCl2	3.0

H^2^O	10.0

10× PCR Buffer	2.5

Taq DNA Polymerase	0.5

**Total**	22.5**
** Add 20 μl allows for pipetting error	

Reaction cocktails for selective PCR amplifications of the ligated DNA	

**Table 5:** Thermocycler conditions for selective PCR.

**Selective Cycles**			
**Step**	**Temperature**	**Duration**	**Number of Cycles**

Initial Denaturation	94°C	120 sec	1

			

Denaturation	94°C	50 sec	

Annealing	56°C	60 sec	2

Extension	72°C	60 sec	

			

Denaturation	94°C	50 sec	

Annealing	56°C	60 sec	20

Extension	72°C	120 sec	

			

Final Extension	72°C	10 min	1

Diluted selective amplification products were purified using fine G-50 Sephadex (Sigma-Aldrich Corp.). We loaded 1.5 μl of purified product per sample along with 0.5 μl GeneScan-500 ROX ladder (PerkinElmer, Inc.) into 96-well plates. Samples were analyzed using an automated ABI 3100 DNA sequencer (Applied Biosystems) and electropherograms were imported into GeneMarker v. 1.6 (SoftGenetics, LLC.) for analyses.

### Fragment Analysis

**Figure 2:**
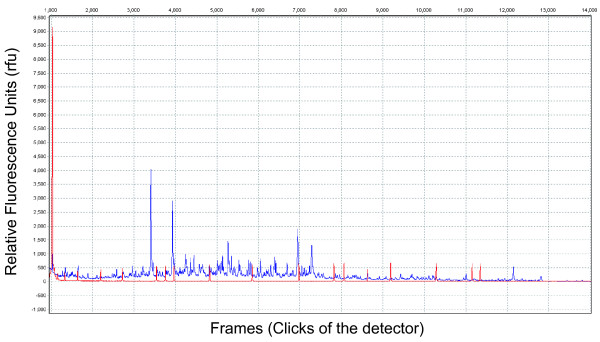
**An example of a raw AFLP data electropherogram**. The y-axis is intensity of the peak measured in relative fluorescence units (rfu) and the x-axis is clicks of the ABI 3100 detector in frames. Blue peaks are the AFLP fragment data and the red peaks are the ROX size standard.

Using GeneMarker v. 1.6, intensity of peaks in the raw data were normalized, without application of the size standard, by generating a template with the AFLP signal processed between 1200 and 11,500 relative fluorescence units (rfu). For the raw data analysis, local southern size-call algorithm, peak saturation, baseline subtraction, pull-up correction, and spike removal correction were selected (Figure 2). Following normalization, allele call was performed with application of the size standard.

**Figure 3:**
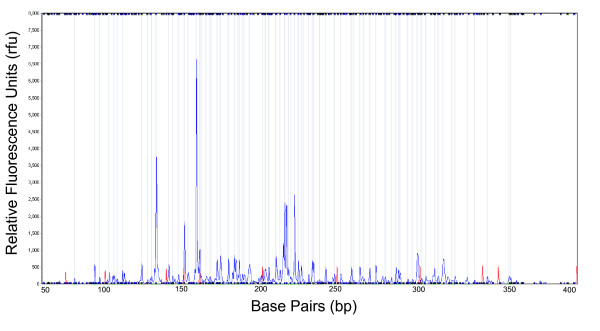
**An example of allele-call data**. The y-axis is intensity of the peak measured in relative fluorescence units (rfu) and the x-axis is the number of base pairs (bp). The gray lines indicate which peaks were scored as present (1) in GeneMarker v. 1.6. Blue peaks are the AFLP data and the red peaks are the ROX size standard.

For allele-called data, only electropherograms in which the size standard used in the analysis matched a theoretical standard by 90% or greater were included for further analysis. A peak was considered an allele if peak intensity was between 100 – 8000 rfu and peaks were longer than 60 bp, which was the shortest fragment length in which clearly defined peaks appeared (Figure 3). However, other researchers have suggested that fragments should only be considered alleles with minimum lengths of 75 bp [19] and 125 bp [20] due to an increased probability of homoplasy. Allele-called data was standardized across individuals for each primer combination by creating a unique standardizing panel (i.e., panel editor in GeneMarker v. 1.6). To generate a standardizing panel, we chose 10 individuals from discrete populations that exhibited the greatest polymorphism (i.e., greatest number and largest range of peaks). Allele positions within templates were further standardized by setting the range around an allele as ± 0.4 bp (i.e., bin size in GeneMarker v. 1.6). Thus, two fragments that fell within this range would be considered one fragment. Creation of a standardizing template for each primer combination ensured that peak position (a peak is equivalent to an allele) was precise for all electropherograms.

After normalization, a binary matrix was generated for each primer combination. An allele was denoted as "1" if the peak intensity was greater than 100 rfu and the fragment occurred between 60 and 350 bp. An allele was considered as "0" if these conditions were not met. Any questionable alleles were manually checked and scored accordingly. All matrices were combined to form one binary matrix for further analyses.

### Repeatability

To test repeatability of replicated samples within and among 96-well plates, PAUP* v. 4.0b10 [21] was used to generate phylogenetic hypotheses using the minimum-evolution procedure (total character and Nei-Li [22] distance options were selected). For each primer combination, replicated samples were placed within a binary matrix with an equal number of non-replicated samples. Repeatability was achieved if replicate samples were most closely related to each other.

## Results and discussion

We generated AFLP fragments for two unrelated taxa using a modified AFLP protocol of Vos et al. [9] and Berres et al. [15]. Our data support findings in the literature that the quality of DNA is more important than the initial concentration [11] as we successfully recovered alleles of samples that varied in DNA concentration from 5 to 70 ng/μl. It is necessary to visually check (i.e., on a gel) the quality of the DNA to ensure that minimal degradation has occurred if samples are stored at -20°C for more than a few weeks in a 0.5 μl PCR tube.

**Figure 4:**
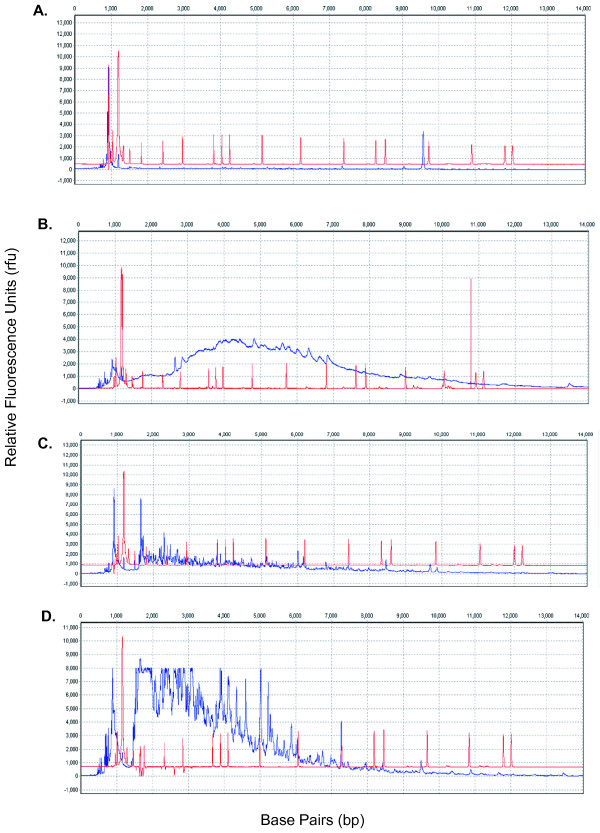
**Examples of poor quality raw AFLP electropherograms**. In most cases, the only solution is to run PCR again. A. An example of the AFLP electropherogram in which the AFLP data (as indicated by the blue line) is too low and is located below the size standard (the red line). The peak intensity is too low and does not permit confident scoring. B. In this example, the peak intensity forms a hill and should not be scored. C. During this run, as can be seen by the electropherogram, the analysis stopped working around 6000 frames. D. The peak intensity is too high and has saturated the ABI 3100. The saturation point for the ABI 3100 is 8000 relative fluorescence units (rfu). The peaks that are squared off at the 8000 rfu point cannot be confidently scored.

During various stages of AFLP generation, DNA concentration may be too high and, if left undiluted, the automated sequencer will become saturated. This will result in peaks that are squared-off at the apex and do not reach the baseline (Figure 4d). In order to prevent saturation, gel images are essential to determine the level of dilution, if any, for each sample; we found that the dilution amount varied between our taxa. We found that after preselective PCR for salamanders, samples were diluted to one part PCR product to three parts water, and for snails, samples were diluted to one part PCR product to five parts water. For other unrelated taxa, researchers may need to further modify concentrations to achieve optimal datasets as shown in Figures 2 and 3.

**Figure 5:**
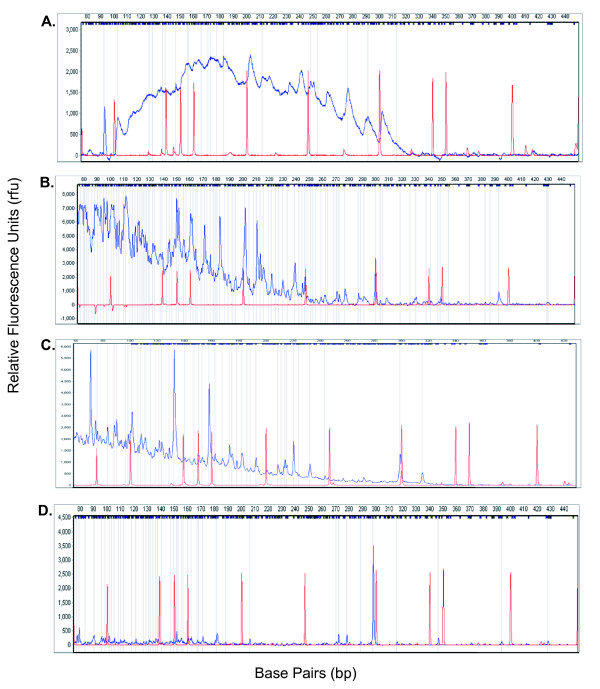
**Examples of poor quality allele call AFLP electropherograms**. In most cases, the only solution is to run PCR again. A. Some alleles identified as peaks can be called in this example. However, many alleles will go undetected because of the structure of the electropherogram. B. The ABI 3100 was saturated in the beginning of the run and the remaining portion of the run is not complete. C. The reaction stopped working and many alleles will not be automatically called by the allele-calling software. D. The peak intensity is too low for many alleles to be called as present.

Some samples meeting all methodological requirements still yielded poor quality electropherograms and were excluded from analyses (Figures 4 and 5). Generally, we found that normalization of raw data using techniques such as pull-up correction, baseline subtraction, peak saturation, smoothing, spike removal, and local southern size call were sufficient to normalize peak intensity across all electropherograms. If normalization is not successful, peaks will extend either below the size standard (Figure 4a) or above the saturation point of 8000 rfu (Figure 4d). If this occurs, even after implementation of the normalization procedure, the researcher must repeat PCR stages for any failed samples.

After raw data were successfully normalized (Figure 2), electropherograms were standardized using the panel editor. This allowed for precise calling of alleles across all samples for each primer combination (Figure 5). Generally, most samples conformed to successful allele calling; however, it is essential that each sample is manually checked to ensure alleles are properly called. Regardless of the quality of the electropherogram, there will be peaks that cannot confidently called by the software (denoted by a '?' in GeneMarker v. 1.6). In this case, each questionable peak must be manually checked and subsequently scored.

Repeatability is essential for the construction of phylogenetic hypotheses using AFLP fragments [8]. The use of a few replicated samples from a 96-well plate allows for cost-effective and reliable tests of repeatability. We were able to generate lineages that contain replicate samples using the minimum evolution procedure. Nodes containing only individual replicate samples were formed in every case for all primer combinations (results not shown). In addition, we calculated a 10% threshold value, which we define as the percentage of allelic differences between replicate samples, in which replicate samples did not form distinct lineages. This threshold value is markedly greater than expected. Although not used in this study, researchers may want to consider the use of the multiple dyes feature available with automated capillary systems. Because this methodology allows for the simultaneous analysis of several primer combinations, it is a cost-effective, efficient means to generate a large number of AFLP fragments while reducing scoring errors that may be associated with batch effects.

## Conclusion

Nuclear markers generated by the cost-effective AFLP technique are broadly used in genetic studies across many taxa regardless of the size or complexity of the genome. We presented a modified protocol that generates AFLP fragments in snails and salamanders, with options for refining this technique to suit the needs of most researchers. Refinement of existing AFLP protocols was essential to facilitate and encourage the broader use of AFLP fragments in genetic studies in animal taxa. Our protocol provides a starting point for researchers to use AFLP, in studies of natural animal population regardless of their taxonomic status and genomic complexity.

## Competing interests

The authors declare that they have no competing interests.

## Authors' contributions

JAW and LTJ contributed equally to all parts of methodological development and manuscript preparation. Both authors read and approved the final manuscript.
